# Complexity Matters: On Gender Agreement in Heritage Scandinavian

**DOI:** 10.3389/fpsyg.2015.01842

**Published:** 2015-12-18

**Authors:** Janne Bondi Johannessen, Ida Larsson

**Affiliations:** ^1^MultiLing and Department of Linguistics and Nordic Studies, University of OsloOslo, Norway; ^2^Department of Linguistics and Nordic Studies, University of OsloOslo, Norway

**Keywords:** Norwegian heritage language, Swedish heritage language, complexity, noun phrase, agreement, gender, declension class, attrition

## Abstract

This paper investigates aspects of the noun phrase from a Scandinavian heritage language perspective, with an emphasis on noun phrase-internal gender agreement and noun declension. Our results are somewhat surprising compared with earlier research: We find that noun phrase-internal agreement for the most part is rather stable. To the extent that we find attrition, it affects agreement in the noun phrase, but not the declension of the noun. We discuss whether this means that gender is lost and has been reduced to a pure declension class, or whether gender is retained. We argue that gender is actually retained in these heritage speakers. One argument for this is that the speakers who lack agreement in complex noun phrases, have agreement intact in simpler phrases. We have thus found that the complexity of the noun phrase is crucial for some speakers. However, among the heritage speakers we also find considerable inter-individual variation, and different speakers can have partly different systems.

## Introduction

As has been shown in a number of recent studies (e.g., Montrul, 2008; Pascual y Cabo et al., 2012; Benmamoun et al., 2013; Kupisch et al., 2014; Johannessen and Salmons, 2015; Polinsky, 2015b; Larsson and Johannessen, 2015a,b), heritage languages can provide important insights into the nature of language acquisition, the linguistic effects of bilingualism across the lifespan of the speaker, and the principles behind linguistic change^1^. In earlier work (Larsson and Johannessen, 2015a,b; Larsson et al., 2015), we have identified four different factors that affect the development of the Scandinavian heritage language in America: contact between Scandinavian and English, contact between Scandinavian dialects (leading to dialect leveling and koineization), incomplete acquisition due to limited input and a language shift around the time of school start, and attrition. Attrition here refers to the loss of linguistic abilities that were once present in the speaker, due to lack of language use. Incomplete acquisition, on the other hand, refers to changes between generations, where the new generation acquires a grammar that is different in some respect from the grammar of the parents, due to limited or conflicting input (cf. Montrul, 2008, and see e.g., Sorace, 2004 who argues that it is important to distinguish what is lost in the language of the individual from features that were never there)^2^.

These factors appear to play different roles in different linguistic domains. For instance, it has been shown that direct transfer from English affects the vocabulary (including function words), but not necessarily core syntax (Haugen, 1953; Hasselmo, 1974; Johannessen and Laake, 2012, forthcoming; Larsson et al., 2015). Larsson and Johannessen (2015a,b) argue that incomplete acquisition on the other hand has led to syntactic change: Heritage Scandinavian has a different word order in embedded clauses than do the Norwegian and Swedish varieties as spoken in Scandinavia. Attrition, we have argued, might, on the other hand, lead to loss of verb second in root clauses in some speakers (Eide and Hjelde, 2012; Johannessen, 2015a; Larsson and Johannessen, 2015b). The two syntactic changes thus seem to have different sources. In the former case, the adult heritage speakers pattern with pre-school L1 children; embedded word order is known to be difficult in L1 acquisition, too (see Larsson and Johannessen, 2015b and references there). In the latter case, the heritage speakers do not necessarily pattern with L1 learners, and the change is restricted to speakers that have not used their language regularly for many years, and who show other signs of attrition (most evidently, lexical retrieval delays). However, change that is due to attrition can be difficult to distinguish from change that should be understood in terms of incomplete acquisition, and it is likely that the two can be interrelated in individual speakers, and have similar results. There are differences, though. Attrition is expected to affect speakers that do not use their first language, regardless of the context of acquisition: Both heritage speakers and immigrant speakers can be affected equally. Among the Heritage Scandinavian speakers, we expect that incomplete acquisition affects most speakers in the community, since they typically have a very similar context of acquisition, but can have different patterns of language usage later in life. Incomplete acquisition is also expected to affect deeper grammatical properties in a different way than attrition, which most clearly affects processing and lexical retrieval (cf. Montrul, 2008).

In this paper, we look closer at one linguistic domain, the noun phrase. Scandinavian noun phrases clearly pose several difficulties in language acquisition, including double definiteness marking, agreement and gender assignment to the noun, and it has previously been shown to be affected in attrition. In a study of five young expatriate Swedes who had not spoken Swedish since childhood, Håkansson (1995) observed deviations in noun phrase–internal agreement in 35–68% (depending on the speaker) of the noun phrases, while word order (verb placement in main and embedded clauses) was target-like. In this respect, these heritage speakers behave differently from both L1 and L2 learners of Swedish; the latter typically show more deviations in word order than in morphology (see e.g., Pienemann and Håkansson, 1999). Other studies have confirmed that morphology is more sensitive to attrition than syntax. For instance, it has been suggested specifically that gender in Heritage Norwegian is being attrited (Lohndal and Westergaard, 2014).

Here, we take a look at noun phrase agreement in Heritage Scandinavian speakers in America, with some comparison with immigrant speakers^3^. We focus on noun phrase internal agreement but also discuss noun declension. We will see that different groups of speakers produce deviations (relative to a baseline) in agreement, but few (if any) deviations with regard to declension class. We will also look at which forms are used, and what the reason for the deviations might be.

The paper is outlined as follows. Section Nominal Agreement and Declension Class in European Norwegian and Swedish gives an overview the relevant aspects of Scandinavian noun phrase morphosyntax and establishes a baseline. In Section Nominal Agreement and Declension Class in Heritage Norwegian and Heritage Swedish, we investigate American Norwegian and American Swedish respectively. In Section Results and Discussion, we discuss the patterns that we can observe with respect to differences between determiners and adjectives, the morphological forms that are used, and the role of complexity. We also briefly comment on the issue of attrition and acquisition. Section Conclusion gives the conclusion.

## Nominal agreement and declension class in european norwegian and swedish

In this section, we give a brief overview of noun phrase morphosyntax in European Norwegian and Swedish, which we assume, based on a cursory study of old recordings of American Norwegian and American Swedish, is the same in relevant respects to the language of the early immigrants (cf. Larsson and Johannessen, 2015b). This will form the baseline for our research on Heritage Scandinavian. When we discuss deviations in the language of the heritage speakers, these are understood in relation to the baseline. However, we maintain a rather liberal view of the baseline language, and only treat something as a deviation if the examples do not occur as dialect forms. In this way, we by necessity include in the baseline what was possibly present in the input of the first generation American-born heritage speakers, rather than what we know was in the input of our speakers^4^.

There is considerable variation in nominal morphosyntax across the Scandinavian dialect continuum, for instance in the distribution of determiners, agreement on predicative adjectives, and in the gender system, some of which is relevant for the present study and will be discussed below. Importantly, it is possible to make generalizations that cover the Norwegian and Swedish varieties, and the variation that can be found is neither random nor unrestricted. Moreover, we have some knowledge of the dialect background of the heritage speakers in the study, and available dialect material (in particular the Nordic Dialect Corpus, Johannessen et al., 2009) makes it possible for us to check the American-Scandinavian data in relation to dialect speakers in Scandinavia. In the overview, we focus on the general features of Norwegian and Swedish nominal morphosyntax. (See Julien, 2005 for a thorough discussion of Scandinavian noun phrases, and Vangsnes et al., 2003 and Dahl, 2015 on dialect variation).

### Determiners, adjectives, and agreement

Both Norwegian and Swedish have definite and indefinite articles (determiners), and there is a definiteness suffix (in addition to the prenominal definite article; see below on double definiteness). Determiners and adjectives are generally prenominal in both Norwegian and Swedish, see (1). Possessive pronouns are found both pre- and post-nominally depending on variety, but post-nominal possessives are infrequent in present-day Swedish. In Norwegian and Swedish, attributive adjectives, and determiners are inflected for number, definiteness, and, in indefinite singular noun phrases, for gender, as in (1). The system is represented by Norwegian in this section.

(1)    a.    *et*       *gammel-t hus*    (Norwegian)

               a.n.sg old.n.sg   house

         b.   *en        gammel-Ø hest*

               an.m.sg old.m.sg   horse

               ‘an old horse’

         c.   *ei       lit-a        elv*

               a.f.sg little.f.sg river

               ‘a small river’

In the present study, we focus on noun declension and noun phrase-internal gender agreement. Since some dialects lack predicative agreement (see e.g., Sandøy, 1988), predicatives are not considered here. In (2–4), we illustrate the agreement paradigms for determiners and adjectives. Note that in the Norwegian and Swedish adjectival paradigm, as in other Germanic languages, a distinction is made between so-called weak and strong inflection. Weak adjectival inflection is used in definite noun phrases, (2), and, in Mainland Scandinavian, does not distinguish gender and number. In this study, only strong inflection of adjectives is included, in addition to singular determiners, which always show gender inflection.

(2)    a.    *den              gaml-e  ku-a*           (Norwegian)

               the.f.sg. def old.def cow.f.sg.def

               ‘the old cow’

         b.   *den               gaml-e hest-en*

               the.m.sg. def old.def horse.m.sg.def

               ‘the old horse’

         c.    *det            gaml-e hus-et*

               the.n.sg.def old.def house.n.sg.def

               ‘the old house’

         d.    *de          gaml-e hest-ene*

               the.pl.def old.pl horse.pl.def

               ‘the old horses’

Notice also that definiteness is marked in more than one place in all of the noun phrases in (2): both on the pre-posed definite article and on the nominal suffix. This way of marking definiteness is usually called double definiteness, and it distinguishes Norwegian and Swedish from e.g., Danish. Most definite noun phrases that have adjectival modification require double definiteness in Norwegian and Swedish, but the pre-posed article is otherwise not used in definite noun phrases; cf. (3) where the pre-posed article is not required (and, in fact, not possible unless the noun is modified by a relative clause or a preposition phrase).

(3)    a.    *ku-a*           (Norwegian)

               cow.f.sg.def

               ‘the cow’

         b.   *hest-en*

               horse.m.sg.def

               ‘the horse’

         c.   *hus-et*

               house.n.sg.def

               ‘the house’

Strong adjectival inflection appears in indefinite noun phrases. Gender is marked in the singular as in (1), but not in the plural (4).

(4)    a.    *gaml-e hest-er*   (Norwegian)

               old.pl horse.pl

               ‘old horses’

         b.   *gaml-e hus*-Ø

               old.pl house.pl

               ‘old houses’

The Standard Swedish and Norwegian (Bokmål^5^) paradigms for the indefinite and the pre-posed definite article are given in Table 1 below. The paradigm for adjectives is given in Table 2.

**Table 1:** **Definite and indefinite articles (determiners) in Norwegian (Bokmål) and Swedish**.

	**M.SG**	**F.SG**	**N.SG**	**PL**
Indef.	No. *en*	*ei*	*et*	Ø
	Sw. *en* (common gender)		*ett*	Ø
Def.	*den*		*det*	*de*

**Table 2:** **Adjectival inflection in Norwegian and Swedish**.

	**M.SG**	**F.SG**	**N.SG**	**PL**
Indef. (“strong” inflection)	No. Ø	-a^*^	-*t*	-*e*
	Sw. Ø (common gender)		-*t*	-*a*
Def. (“weak” inflection)	No. -*e* (for all genders and numbers)			
	Sw. -*a* (for all genders and numbers)			

* The Norwegian indefinite singular adjectival inflection is very rare, and only exists for a handful of lexemes.

The only differences between different varieties of Swedish and Norwegian lie in the presence/absence of the feminine, and in the fact that some varieties have the suffix -*e* (like No. Bokmål) while other varieties (like Standard Swedish) have -*a* on definite or plural adjectives^6^.

Our cursory study of the older American Scandinavian recordings show that there is (as expected; cf. Section Gender below) some variation in the use of the feminine forms, and differences in the distribution of determiners (which are irrelevant for our purposes). Overall, the system is however identical to the system outlined above. We will therefore use this system as a baseline. If anything, the older heritage language has more morphological distinctions within the noun phrase than we have provided here, such as dative morphology (Johannessen and Laake, 2012), and not less. If we find examples of bare stems rather than inflected words, we therefore know that this is a deviation from the baseline.

### Gender

In the following, we take gender to be an agreement category, distinct from declension class (following e.g., Corbett, 1991), but we make a distinction between gender agreement and gender assignment to the noun. In the latter, gender is generally assumed to be an inherent (lexical) property of nouns (e.g. Julien, 2005), but cf. e.g., Nygård and Åfarli (2013) who argue that gender is assigned to the noun in the syntax. Gender assignment is generally semantically opaque in Norwegian and Swedish, and it often has to be learned for individual lexical items (see e.g., Trosterud, 2001; Enger, 2014 for discussion).

The old Germanic three-gender system is retained to a lesser or higher degree. In Standard Swedish and some Norwegian varieties, the masculine and the feminine have collapsed into a common gender (see Fretheim, 1976/1985; Lødrup, 2011; Trudgill, 2013 for discussion). In many such varieties, feminine forms are retained in the nominal declension only, with no feminine features on adjectives or determiners^7^. Instead, these nouns must be considered masculine/common gender, since their determiners and adjectival modifiers follow the masculine/common gender agreement pattern. In varieties with three-gender agreement, the indefinite determiner can be inflected in the feminine, as can a handful of adjectives (depending on variety), as exemplified in (1c). In varieties that only have two genders, the traditionally feminine nouns agree like (5), which is parallel to (1b):

(5)    *en      lit-en      elv*   (Norwegian)

        a.m.sg little.m.sg river

        ‘a little river’

Even in varieties of Norwegian and Swedish that retain the feminine, there can be a tendency toward a two-gender system, in which neuter remains as before, while masculine forms takes over at the expense of feminine forms.

If the Scandinavian Heritage language speakers have simplified their gender assignment system toward a default gender, it is likely that it will be toward the masculine. There are several reasons for this. First, the masculine/common gender is morpho-phonologically less marked than neuter. Both languages have Ø marking on the strong adjectival masculine inflection, while neuter singular has the suffix –*t*. Secondly, masculine nouns are more frequent than feminine nouns in the dialects that have the feminine (both with respect to type and token frequency; for Norwegian, see Heggstad, 1982, p. 12)^8^. In Swedish, common gender nouns are more frequent than neuter nouns (cf. Källström, 2008). More importantly, in the present context, masculine is the most popular gender for loanwords in the American Scandinavian varieties: for instance around 70% of the loan words in American Trønder^9^ Norwegian are masculine (Hjelde, 1992, p. 84). Additionally, the masculine/common gender is also often overgeneralized in first and second language acquisition (see e.g., Rodina and Westergaard, 2013). Masculine has also been generalized in varieties that have changed from a three to a two-gender system. Moreover, in Swedish and Norwegian dialects that lack predicative agreement morphology in the plural, it is the masculine/common gender form that is used with plural predicative adjectives (Larsen and Stoltz, 1911, p. 45; Josefsson, 2009). Determiners like No. *sånn* ‘such’, which in some varieties do not have to agree when they have an abstract modal meaning, have the masculine agreement pattern as their default pattern (Johannessen, 2012)^10^.

### Declension class and gender

In noun inflection, gender is visible on the form of the definiteness suffix (which has historically developed from a determiner) in the singular. In many varieties with a traditional three-gender system, nouns that have -*et* as a definiteness suffix are always neuter, while -*en* is masculine and -*a* feminine. In two-gendered Norwegian and Swedish, there are two general possibilities: In some varieties, -*et* is neuter, and -*en* and -*a* are common gender, whereas other varieties (such as Standard Swedish) has -*et* for neuter and -*en* for common gender. In language acquisition, the definiteness suffix in this way gives an unambiguous clue to the gender of the noun.

One complication is variation in the noun declensions of some old feminine nouns, as in (6). The noun in (6a) has a traditionally feminine form, but it cannot be considered feminine if it only triggers masculine/common gender agreement on determiners and adjectives. In such cases we will use the term declension class, and say that definite nouns ending in -*a* belong to a declension class that allows the -*a* suffix, and not to the feminine gender. We will take both forms to be target-like. (See Faarlund et al., 1997, p. 151 for more on the alternatives that exist.)

(6)    a.    *elv-a*                                        (Norwegian)

               river.m.sg.def (still f.sg.def in many dialects)

         b.   *elv-en*

               river.m.sg.def

In the plural, number inflection only partly reflects gender: Indefinite neuter nouns can have Ø-plural inflection in Swedish and Norwegian, and generally only old feminine nouns have–*or* as the plural suffix in Swedish (but not all do). That is, noun declension might give clues for gender in the acquisitional process, but plural suffixes do not necessarily reveal the gender of their hosts (see Källström, 1996; Enger, 2004 for discussion).

We will use the singular definiteness suffix as evidence for the gender of the noun in the heritage languages as long as we also find gender agreement morphology in the noun phrase. Thus, we follow Enger (2004) and assume that while there is in principle a distinction between gender (which necessarily affects associated words) and declension class, “it would be unwise to claim that the definite singular suffix is exclusively an exponent of declension and not at all of gender” (2004:65) in many varieties of Norwegian and Swedish, since this would leave several generalizations unexplained.^11^ At the same time, an important part of the present study is to determine, for speakers whose agreement patterns are dwindling, whether target noun declension simply signals a declension class, or whether they indeed have gender distinctions, but that attrition has caused deviant agreement patterns in their production (due possibly to processing problems related to lack of practice). The connection between gender and declension class will be discussed further in Section Gender, Agreement, and Declension Class.

## Nominal agreement and declension class in heritage norwegian and heritage swedish

In this study we focus on gender agreement. By *agreement* we mean cases where there is one or more exponents (possibly -Ø) of gender in the noun phrase apart from the noun (cf. e.g., Corbett, 1991; Baker, 2008). The examples we provided in Section Determiners, Adjectives, and Agreement above therefore illustrate gender agreement in Swedish and Norwegian^12^. We investigate attributive agreement inflection on determiners and adjectives as well as forms of the definiteness suffix in the singular. This means that we look at the presence or absence of suffixes that show agreement or non-agreement, as well as aspects of the nominal inflection. In noun phrases where more than one element would show gender agreement in the baseline (e.g., where both adjectives and determiners show agreement), all potentially agreeing forms have been counted separately, and these noun phrases are therefore represented more than once in the numbers.

If an adjective seemingly (given the baseline) does not agree with a suffixless noun, two analyses are possible: Either the adjective does not agree, or the noun has been assigned a different gender in that particular idiolect than in the baseline. The constructed examples in (7) illustrate this. (7a) will be interpreted as a deviation from the baseline with regard to agreement, since different choices have been made in the determiner and the adjective, while (7b) can also be interpreted as involving deviant gender assignment to the noun—unless we find evidence to the contrary—since there is agreement between the first two words, and the suffix-less noun does not reveal its gender. We treated these ambiguous cases separately at the onset. However, as we will see there is reason to assume that (7b), too, involves deviant agreement, given examples like (7c), where the noun is inflected, revealing its gender.

(7)                                                                             (Norwegian)

         a.    *en*         *god-t*                   *gutt* (target: *god* ‘good’.m.sg.)

               a.m.sg   good.n.sg            boy. m.sg.

(target: m.sg., but gender not visible on indefinite nouns)

               ‘a nice boy’

         b.   *et*           *god-t*                  *gutt* (target: *en*. ‘a’ m.sg, *god* ‘good’.m.sg.)

               a.n.sg     good.n.sg          boy.m or n?.sg

(target: m.sg., but gender not visible on indefinite nouns. The speaker seems to have chosen n.sg in the agreeing words, thereby possibly having non-target gender assignment.)

               ‘a nice boy’

         c.    *det           god-e*               *gutt-en* (target: *den* ‘the’.m.sg.def)

               the.n.sg.def good.def       boy.m.sg.def

(target: m.sg., the target gender visible on the suffix of definite nouns)

               ‘the nice boy’

Closer investigation, considering inflected nouns in other sentences, reveals that the heritage speakers in fact do have the standard gender for those nouns. We therefore treat these as deviations in agreement morphology.

We have used partly different methods in the investigation of the Swedish and the Norwegian data, given the different types of data available. The Heritage Norwegian data are transcribed and available in a corpus (of 34 speakers), inviting more quantitative data (in addition to a closer study of two speakers). For Swedish, we have selected eight speakers with different backgrounds, but with Swedish as (one of) their L1^13^. We think that this combination of quantitative and qualitative data is possible since Swedish and Norwegian grammar share many of the relevant features. The different methods also contribute to the study in different ways, thus strengthening the results. The material is arguably rather small, but as we will see, we can still observe some patterns in the inter-individual variation, by looking in more detail at the individual speakers.

### Agreement in the corpus of american norwegian speech

The speakers in the Corpus of American Norwegian Speech (CANS, Johannessen, 2015b) are all born in the USA between the years 1900–1940. None of these speakers use their L1 very often, and they are all expected to show some signs of attrition (like lexical retrieval delays), in addition to changes due to incomplete acquisition or koinéization (cf. the introduction). (Due to the high number of speakers, we will not give more detailed information on these, except two of them in Section A Closer Look at Two of the Heritage Norwegian Speakers, unlike what we do for the Swedish heritage speakers in Section Heritage Swedish.)

We searched for the combination of (determiner)—adjective—noun in the corpus.

There are 171 hits of noun phrases where gender agreement is relevant. These are divided between 58 with the sequence adjective–noun (excluding those with a pre-adjectival determiner) and 113 cases for the sequence determiner–adjective–noun. There are altogether 21 cases that have non-target-like adjective or determiner agreement, but none that has a deviant definiteness suffix, see Table 3 below.

**Table 3:** **Gender agreement and the definiteness suffix in Heritage Norwegian**.

	**Non-target agreement**	**Non-target definiteness suffix**
Adjective-noun sequence	1/58 (2%)	0/58 (0%)
Determiner-adjective-noun seq.	20/113 (18%)	0/113 (0%)
Total	21/171 **(12%)**	0/171 **(0%)**

There is a substantial difference in performance between the adjective-noun sequence and the determiner-adjective-noun sequence. (And notice that in the latter there can be non-target use of either of the two categories determiner and adjective, see Sections A Closer Look at Two of the Heritage Norwegian Speakers and Determiners and Adjectives) In the rest of the paper, we will refer to the combination of determiner-adjective-noun (sometimes with an omitted head noun, however) as a complex noun phrase, and to those with determiner-noun or adjective-noun as simple. The terms are here understood in a pre-theoretical sense—relating to the linear string, not hierarchical structure (see further Section Gender, Agreement, and Declension Class for discussion). As we shall see later, there are two different interpretations of the data in Table 3. Either noun phrases with determiners are more difficult, or it is complexity that matters. In the following sections we include determiner–noun sequences to investigate the two possibilities.

The complex noun phrases have 18% deviant constructions amongst all the speakers; a relatively high number. Some examples are provided below in (8):

(8)                                                (Heritage Norwegian)

         a.    *en                   fin-t*

               a.m.sg.indef nice.n.sg.indef

                  *maskin* (target: *fin* ‘nice.m.sg.indef)

                  machine.m.sg.indef

               ‘a nice machine’ (Rushford_MN_01gm)

         b.    *ei                   stor*

               a.f. sg.indef big.f/m. sg.indef

                     *famili* (target: *en* ‘a.m.sg.indef)

                     family.m.sg.indef

               ‘a big family’ (Harmony_MN_02gk)

         c.    *denna            andre         skolehuset*

               this.m.sg.def other.def school.building.n.sg.def

                       (target: *detta* 'this.n.sg.def)

               ‘this other school building' (westby_WI_06gm)

In (8a), it is the gender on the adjective that deviates: the neuter form is used for the masculine (*fin*). In (8b) and (8c), it is the gender of the determiner that deviates from the baseline. In (8b) the feminine form of the indefinite article is used for the masculine (*en*). In (8c), the masculine form of the demonstrative (*denna*) is used for the expected neuter (*detta*)^14^.

From the examples in (8) we see that the deviations involve several different forms, and that both adjectives and determiners can deviate. Deviant agreement can occur on determiner, adjective or both. However, there are some patterns. First, there are more deviations in determiners than in adjectives (cf. Section Determiners and Adjectives). Secondly, the neuter definite determiner *det* for the expected M/F determiner (or demonstrative) *den* occurs six times, whereas *den* is used twice for the neuter *det*. The feminine indefinite determiner *ei* is used twice for the expected masculine, and the masculine indefinite determiner is used three times in a neuter context. Thus, we cannot observe any clear generalization of the default in this data set, except that the non-target neuter definite singular determiner is used more often than the others. With respect to deviating adjectives, the picture is somewhat different: the masculine (corresponding to the bare stem) occurs for neuter six times, whereas neuter is used for M/F three times.

We have seen that Heritage Norwegian basically has target-like agreement, though there is 12% deviance in the relevant constructions. Looking closer at the speakers, however, it quickly becomes evident that there is considerable inter-individual variation. In fact, the majority of the speakers (20/34) in the CANS corpus only produce target-like examples, while 14/34 (41%) also produce non-targetlike examples. These speakers produce 86% of the hits, which means that while they make errors, they also produce a lot of utterances. There is also much individual variation in the frequency of non-targetlike NPs among the speakers that show deviations, ranging from 6% (3/52; coon_valley_WI_06gm) to 38% (5/13; Harmony_MN_02gk). This means that while many of the speakers in the corpus have baseline command of gender agreement, not all do. As a comparison, the adult Norwegian speakers (age 31–64) in Rodina and Westergaard (2015) show an accuracy of 99–100%.

It seems that for those speakers that produce non-target noun phrases, agreement poses problems, while gender assignment to nouns, as apparent from the definiteness suffix, is unproblematic. As noted above, examples like (9) could in principle be instances of an idiosyncratic gender assignment by the speakers, in which both *bilde* and *farmeår* are masculine rather than the target neuter. But the inflected forms of the noun å*r* ‘year’ in the CANS corpus reveal that this word is rarely treated as masculine. The corpus gives 24 hits for the neuter å*ret* ‘year.n.sg.def’, and none for å*ren* ‘year.m.sg.def’. Searching for a simple noun phrase like *ett år* ‘one year’, we get 19 hits with a neuter determiner (*ett*, æ*it, itt* etc.), and 8 with a masculine determiner (*ein, en, enn*). So both the definiteness suffix and the indefinite determiner reveal correct agreement forms compatible with the neuter-ness of the noun. Our hypothesis is that it is agreement rather than gender assignment and declension class that deviates for the heritage speakers. It is therefore worth asking if it is the complexity of the noun phrase that determines the extent of agreement.

(9)   a.   en   god       farmeår

             a.m good.m farming.year.n

                    (target: *et* 'a'.n *godt* 'good'.n)

            'a good farming year' (decorah_IA_02gm)

       b.   en    gammel bilde

             a. m old.m    picture.n

                    (target: *et '*a'.n *gammelt '*old'.n)

             'an old picture' (chicago_IL_01gk)

In the next section, we look closer at the two Norwegian Heritage speakers that have revealed the highest amount of deviant constructions, since these are the ones that are likely to have enough data to be subjected to a quantitative comparison of constructions and for possible systematicity to be evident.

### A closer look at two of the heritage norwegian speakers

In the previous section we saw that amongst the 34 Norwegian heritage speakers in the CANS corpus, most have target-like agreement. Out of those that do not have full target-like agreement, there is great inter-speaker variation. In order to be able to investigate the linguistic properties that show some deviance, we need to look at speakers that produce some quantity of non-target agreement. There are two such speakers, Daisy (Chicago_IL-01gk) and Elsa (Harmony_MN_02gk). Daisy, 89 years old in 2010, was born in 1920 in Chicago by Norwegian immigrants, and Norwegian was spoken alongside English in her childhood home^15^. Her late husband did not speak Norwegian, and neither did her children. However, her father had lived with her until he died 15 years previously. She had not spoken Norwegian since. Daisy had been to Norway on five-six short trips. Elsa was born in 1930 in Spring Grove, all her grandparents had been born in Norway. She did not learn English until she started school. Her husband speaks Norwegian, but they never speak together. Two sons have settled in Norway with Norwegian families. She has been to Norway nine times, and has received several visits.

We can directly note that these two speakers have different gender systems. Whereas Elsa has a typical three-gender system, Daisy from Chicago has a Norwegian town dialect (reflecting, probably, her mother's dialect), and has only two genders: common gender (M/F) and neuter (N). Thus, we find both two-gender and three-gender systems among the speakers that show the most deviations. For these speakers, we have investigated the sequence determiner-adjective-noun, as well as the simpler (in a linear sense) phrases with determiner-noun and noun-possessive. We have also investigated all definite forms of their nouns. If these speakers reveal a difference between simple and more complex constructions, this will take us some way toward understanding the reason for the deviations. Notice that all pre-posed demonstratives, articles and possessives are regarded as determiners (see footnote 14) in Norwegian.

Table 4 sums up the findings for the two Norwegian speakers that have the most occurrences of deviant forms.

**Table 4:** **Noun phrase morphology produced by the two most deviant Heritage Norwegian speakers**.

	**Deviant det-adj-noun agreement**	**Deviant det-noun agreement**	**Deviant noun-postposed poss. agreement**	**Deviant definiteness suffix**
Daisy (Chicago_IL-01gk)	6/12 (50%)	4/51 (8%)	0/30 (0%)	0/59 (0%)
Elsa (Harmony_MN_02gk)	2/4 (50%)	2/23 (9%)	0/10 (0%)	0/39 (0%)
Total	8/16 **(50%)**	6/74 **(8%)**	0/40 **(0%)**	0/98 **(0%)**

The table shows that there is indeed a difference between the more complex structures and the simplest ones, in which the most complex structures have 50% deviant agreement, while the much simpler determiner-noun structures have only 8% deviant forms, and the noun-possessive and noun-suffix structures have no deviance at all. Thus, it is quite clear that complex constructions are a challenge for these heritage speakers (though the absolute numbers are low). This result supports the trend in Table 3, Section Agreement in the Corpus of American Norwegian Speech, even if we have investigated only two speakers (though since the two speakers are also amongst the group of speakers in Table 3, we should not put too much emphasis on this).

In complex noun phrases, five of Daisy's non-target cases fall into the category of a non-target determiner, in which the neuter *det* has been chosen instead of the target masculine *den.* Elsa's non-target cases consist of one case similar to Daisy's, in which the neuter has been chosen for the masculine, and one in which a feminine indefinite article has been chosen instead of a masculine one, *ei* instead of *en*. There is only one non-target gender in the adjective inflection in these complex noun phrases, and there a neuter noun has been modified by a masculine determiner and a masculine adjective. We present complex noun phrases with non-target forms produced by our two informants in (10)^16^.

(10c) was produced twice by Daisy, and once by Elsa. In (10d) there is both a deviant determiner and a deviant adjective.

(10)   a.   *det               siste      plass*

               the.n.sg.def last.def place.m.sg.indef

                       (target: *den* 'the'.m.sg. def)

              'the last place' (chicago_IL_01gk)

          b.  *det               eldste       John*

               the.n.sg.def oldest.def John. m

                       (target: *den* 'the'.m.sg. def)

               'the oldest, John'^17^ (chicago_IL_01gk)

          c.  *det               første    gang*

               the.n.sg.def first.def time.m.sg.indef

                       (target: *den* 'the'.m.sg. def)

              'the first time' (chicago_IL_01gk * 2, harmony_

                       MN_02gk * 1)

          d.  *en                 gammel            bilde*

               a.m.sg.indef old. m.sg.indef picture.n.sg.indef

               'an old picture' (chicago_IL_01gk)

               (target: *et* 'a'.n.sg.indef, *gammelt 'old'*.n.sg. def)

          e.  *ei                 stor                    familie*

               a.f.sg.indef big.m/f.sg.indef family.m.sg.indef

                        (target: *en*.m.sg. indef)

               'a big family' (harmony_MN_02gk)

If we compare the complex noun phrase with a simple noun phrase (determiner–noun), we can see a clear difference, as evident from Table 4. In simple noun phrases, the two speakers achieve the target in the vast majority of the cases. There are 74 relevant cases, and of these 68 (92%) are target-like, i.e., only 8% are non-target-like, due to wrong gender agreement marking. This also shows that it is the complexity of the noun phrase that causes the difficulty with agreement, rather than e.g., difficulty with the forms of determiners *per se*. There are more deviant determiners in the complex noun phrases.

We can note that while we find an alternation between *den* ‘the’.m.sg.def and *det* ‘the’.n.sg.def in both types of noun phrases, the number of non-target *det* is higher in the complex ones. We have, as shown in (12f), only one example of a non-target use of this determiner in simple noun phrases amongst the two speakers. But there are seven examples of the pre-posed, definite determiner *den* ‘the’.m.sg.def that are target-like [see (11)], specifically five by Daisy and two by Elsa. Thus our hypothesis is strengthened; there are possible contexts (in simple noun phrases) where the non-target *det* ‘the’ n.sg.def could have appeared, but does not. (Thanks to a reviewer for this point).

Some target-like examples of the simple determiner-noun sequence are presented in (11). Notice that there is agreement in all three genders (two for Daisy), showing that gender, even for these speakers, is a category that is basically stable.

(11)   a.   *en                 butikk*        (Heritage Norwegian)

               a.m.sg.indef shop.m.sg.indef

               'a shop' (chicago_IL_01gk)

          b.  *den               veien*

               that.m.sg.def way.m.sg.def

               'that way' (harmony_MN_02gk)

         c.   *et                  par                      koner*

               a.n.sg.indef couple.n.sg.indef wives

               'a couple of wives' (chicago_IL_01gk)

         d.   *dette            året*

               this.n.sg.def year.n.sg.def

               'this year' (harmony_MN_02gk)

         e.   *ei                 bok*

               a.f.sg.indef book.f.sg.indef

               'a book' (harmony_MN_02gk)

         f.   *den                kirken*

               the.m.sg.def church.m.sg.def

               'that church' (chicago_IL_01gk)

Since, there are only six non-target determiner-noun sequences with these two speakers, we present all below in (12).

(12)   a.   *ei                brev*

               a.f.sg.indef letter.n.sg.indef

                      (Heritage Norwegian) (target: *et*. n.sg. indef)

               'a letter' (harmony_MN_02gk)

         b.   *ei                 bryllup*

               a.f.sg.indef wedding.n.sg.indef

                      (target: *et*. n.sg.indef)

               'a wedding' (harmony_MN_02gk)

         c.   *en                  fjell*

               a.m.sg.indef mountain.n.sg.indef

                       (target: *et*. n.sg.indef)

               'a mountain' (chicago_IL_01gk)

         d.   *en                  barnebarn*

               a.m.sg. indef grandchild.n.sg.indef

                       (target: *et*. n.sg.indef)

               'a grandchild' (chicago_IL_01gk)

         e.   *det               slags*

               the.n.sg.def kind.m.sg.indef.gen

                        *arbeid*(target: *den*. m.sg.def)^18^

                        work.n.sg.indef

               'the kind' (of work) (chicago_IL_01gk)

         f.   *det               by*               (target: *den*.m.sg.def)

              the.n.sg.def town.m.sg.indef

                 'the town' (chicago_IL_01gk)

Elsa from Harmony has two cases of non-target determiners; in both cases a feminine determiner has replaced the target neuter determiner.^19^ The Chicago informant, Daisy, twice uses the masculine indefinite determiner to replace the neuter one. Conversely, she twice uses the definite neuter determiner *det* /de/ instead of the target masculine *den* /den/. However, she also has some examples of target use of the indefinite neuter determiner (*et par* ‘a pair’ *et hotell* ‘a hotel’), and many examples of target definite masculine determiner, such as *den bygning* ‘the building’, *den dagen* ‘the/that day’. There are several ways to interpret these results. One possibility is that Daisy finds agreement hard and mixes the forms. Another possibility is that she does not have the target gender assignment on the four relevant nouns. The latter can be checked. She uses the word *fjell* in the target definite form *fjellet* ‘the mountain.’ She also uses the correct plural definite determiner that characterizes neuter nouns: *barnebarna* ‘the grandchildren.’ Given, in addition to this, the large number of correct agreement forms, we take it that she does assign the correct gender to her nouns, and she knows basic agreement, but that agreement is sufficiently demanding for her to make non-target performance errors. However, when she chooses the form *det* /de/ instead of *den* /den/, it could have something to do with the phonological and semantic similarity to the English definite determiner *the*. We notice also that there is no hint of a resort to a default masculine gender. (See Section Determiners and Adjectives and Morphological Form and Type of Gender System for more discussion on these matters).

An equally simple pattern is the one with post-posed possessives, i.e., noun-determiner. Here we found 40 relevant hits (removing tagging errors and those where the determiner is invariable, like *hans* ‘his’ and *hennes* ‘her’). In these cases, there was 100% correct score for the two informants. Examples are given in (13)^20^, ^21^.

(13)   a.   *far                       min*  (Heritage Norwegian)

               father.m.sg.indef my.m.sg

               'my father' (chicago_IL_01gk)

         b.   *dattera              mi*

               daughter.f.sg.def my.f.sg

               'my daughter' (chicago_IL_01gk)

         c.   *plassen             din*

               place.m.sg.def your.m.sg

               'your place' (chicago_IL_01gk)

         d.   *mor                     mi*

               mother.f.sg.indef my.f.sg

               'my mother' (harmony_MN_02gk)

Finally, the definiteness suffix is always target-like (see Table 4). Some examples are given in (14).

(14)   a.   *politiskolen*               (Heritage Norwegian)

               police.school.m.sg.def

               'the police school' (chicago_IL_01gk)

         b.   *nabolaget*

               neighborhood.n.sg.def

               'the neighborhood' (chicago_IL_01gk)

         c.   *hytta*

               hut.f.sg.def

               'the hut' (harmony_MN_02gk)

         d.   *attenhundretalet*

               egihteenhundred.period.n.sg.def

               'the 19th century' (harmony_MN_02gk)

### Heritage swedish

The Norwegian data raises a number of questions that we will now address by looking in detail at the Heritage Swedish data. Firstly, some of the inter-speaker variation might be due to what types of utterances the speakers produce. That is, Elsa and Daisy might show more deviations because they produce a higher number of complex noun phrases than other speakers. By considering all utterances produced by a number of Heritage Swedish speakers, we can address this question. Secondly, the corpus data gives us few direct clues to what the reason is that some speakers deviate to a higher or lesser extent. (But we know from a previous study (Johannessen, 2015a) that Daisy shows other signs of attrition.) In the Swedish data, we have access to different groups of speakers—both American-born heritage speakers and immigrant speakers—and by considering their linguistic background, we have a further way of addressing this question, too. Thirdly, by considering more data, and by looking at the inter-speaker patterns, we might find clearer patterns with respect to which forms are overgeneralized and when the deviations occur. We will see that the data from Swedish also supports the conclusion above that deviations involve non-target agreement, rather than non-target gender assignment. Recall that we expect many of the Heritage Swedish speakers to have a two-gender system where the singular definiteness suffix without exception signals the gender of the noun.

Since the background of the speakers is of some importance, we start by giving an overview of this, before we turn to agreement patterns and declension.

#### The heritage swedish speakers

For Swedish we have investigated all noun phrases produced by eight speakers with partly different backgrounds. Two of the speakers (Annie and Martin) are first generation immigrants. In other words, they speak what we could refer to as Immigrant American Swedish. We can assume that they acquired Swedish fully in their childhood. Deviations should therefore be due to attrition rather than incomplete acquisition. The other six speakers are born in the American Midwest. Four of them (Arthur, Albert, Norman, Konrad) were monolingual in Swedish until around the age of 5–6. Of these, Arthur and Albert continued to have Swedish as the dominant language the longest. Albert reports that he grew up with his grandparents who spoke little or no English, and in the fifth grade his teacher told him to start speaking English. Two speakers (Amos and Theodor) report that they were early bilinguals, with both English and Swedish before school start. Annie and Norman are married, and they were interviewed together. A summary is given in Table 5 below.

**Table 5:** **The American-Swedish speakers**.

**Name**	**Born**	**Nationality**	**Speaking pattern**
Annie	1931 in Northern Sweden (Norrbotten)	Emigrated in 1949	Married to Norman, speaks Sw. in some contexts
Martin	1958 in Northern Sweden (Dalarna)	Emigrated in 1968	Speaks Sw. very rarely
Arthur	1929 in Minnesota	Sw. monoling. until school start, much Swedish at home	Grew up with Swedish grandparents, speaks Sw. on occasion
Albert	1921 in Minnesota	Sw. as L1, En. at age ten	Speaks Sw. on occasion
Norman	1930 in Chicago	Sw. monoling. until the age of 5	Married to Annie, hears Sw., speaks rarely
Konrad	1933 in Minnesota	Sw. monoling. until school start	Speaks Sw. very rarely
Theodor	1922	Sw. and En. as L1	Speaks Sw. on occasion
Amos	1921 in Minnesota	Sw. and En. as L1	Has not spoken Swedish since 1976

Like most of the speakers in the American Norwegian corpus, none of the speakers use their Swedish regularly. Amos last spoke Swedish when he visited Sweden in 1976 (36 years ago at the time of the interview). He writes a little Swedish, and we have received some e-mail communication from him. During the interview, Norman is reluctant to speak Swedish, but he has less difficulty after a few minutes. His wife Annie speaks Swedish to some of her friends, and Norman seems more used to listening to Swedish than to speaking it.

Of the two immigrant speakers, Martin has an earlier and more abrupt onset of English than Annie. He emigrated at the age of 10 and reports that he learnt English immediately and without difficulty. He also reports that he no longer speaks Swedish with his parents. At the same time, he behaves like a native speaker in a different way than the American-born heritage speakers. For instance, his judgment of embedded word order is stable and native-like, and on occasion, he uses Swedish derivational morphology productively and correctly to compensate for his lack of vocabulary.

#### Noun declension

We saw above that the Norwegian heritage speakers never have deviations in the form of the singular definiteness suffix. The same is true for the Swedish speakers: Only one of the 201 examples in the data shows a deviation. Some of the target-like examples are given in (15a–d), and the deviating example is given in (15e)^22^. The data is summarized in Table 6.

(15)   a.   *brev-et* (Amos) (Heritage Swedish)

               letter.n.sg.def

         b.   *golv-et* (Arthur)

               floor.n.sg.def

         c.   *konditori-et* (Norman)

               cafe.n.sg.def

         d.   *student-en* (Norman)

               student.c.sg.def

         e.   *krig-en* (Norman)

               war.c.sg.def (target: *krig-et* 'war.n.sg.def')

**Table 6:** **The definite singular form of nouns in Heritage Swedish**.

**Name**	**Non-target definiteness suffix**
Annie	0/22 (0%)
Martin	0/24 (0%)
Arthur	0/13 (0%)
Albert	0/25 (0%)
Norman	1/17 (6%)
Konrad	0/64 (0%)
Theodor	0/13 (0%)
Amos	0/23 (0%)
Total	1/201 **(0.5%)**

However, from this data alone we do not know if the gender system is intact in Heritage Swedish. While the singular definiteness suffix unambiguously signals the gender of the noun in Standard Swedish (also reflected in agreement), it might mark only declension class for these heritage speakers. The Norwegian data suggested that the problem is really agreement, not gender assignment. In the next section, we will see that the same holds for Swedish.

#### Gender agreement in complex and simple noun phrases

We have investigated all noun phrases where gender agreement is expected; the results are summarized in Table 7. As we did for the Norwegian data, we distinguish between complex noun phrases (with determiner-adjective-noun) and simple noun phrases (adjective-noun or more often determiner-noun). The simple noun phrases include two examples with a post-nominal possessive (both produced by Arthur), which is possible in a few Swedish dialects. In principle, many of the deviations in simple noun phrases (and some of the complex ones) are ambiguous between deviating gender agreement and gender assignment. As we have argued for Norwegian, these cases should most likely generally be treated as involving deviating agreement. This will be discussed further below.

**Table 7:** **Gender agreement in noun phrases in Heritage Swedish**.

**Name**	**Non-target agreement in complex noun phrases Det–A–N**	**Non-target agreement in simple noun phrases Det–N or A–N**	**Non-target agreement in total**
Annie	2/45 (4%)	0/24 (0%)	2/69 (3%)
Martin	5/22 (23%)	1/44 (2%)	6/66 (9%)
Arthur	0/13 (0%)	2/17 (12%)	2/30 (7%)
Albert	0/2 (0%)	0/13 (0%)	0/15 (0%)
Norman	6/9 (29%)	1/16 (17%)	7/25 (28%)
Konrad	0/13(0%)	1/81 (1%)	1/94 (1%)
Theodor	4/15 (27%)	3/21 (14%)	7/36 (19%)
Amos	4/11 (36%)	8/24 (33%)	12/35 (34%)
Total	21/130 **(16%)**	16/240 **(7%)**	37/370 **(10%)**

The overall frequency of non-target agreement is 10%, and thus very similar to what we found for Norwegian (12%). As for Norwegian, we find a difference between complex and simple noun phrases: the former show deviations in 16% of the cases, whereas the latter have 7% deviating forms. However, complexity is not a clear factor for all speakers; One speaker (Arthur) has the opposite pattern. Two speakers (Albert and Konrad) are target-like (with a single exception) in both types of noun phrases.

The results reveal considerable inter-individual variation also in other respects. Some speakers show no or almost no examples of deviations, whereas others have a considerable amount. Again, this is what we saw for Norwegian. The variation can give us some insight into the factors that cause the deviant forms. We can note that the two immigrant speakers show partly different behavior. Annie produces only two deviant forms. They occur in the same noun phrase, and this particular example is clearly due to lack of planning: She immediately afterwards switches from the neuter noun *ställe* ‘place’ to non-neuter *by* ‘village’. Martin, on the other hand, shows a few examples of deviations that suggest some occasional difficulty with nominal agreement. In all of these cases, the default (C.SG) is generalized on determiners or adjectives, as in (16):

(16)                                                (Immigrant Swedish)

         a.   *en                  annat*

               an.c.sg.indef other.n.sg.indef

                        *stålverk* (target: *ett* 'an'.n.sg.indef)

                        steelworks.n.sg.indef

               'a different steelworks' (Martin)

         b.   *en                  gammal*

               an.c.sg.indef old.c.sg.indef

                       *stålbruk*

                       steelworks.n.sg.indef

               'an old steelworks' (Martin)

               (target:      *ett*     an.n.sg.indef,   *gammalt*   old.

                        n.sg.indef)

In principle, (16b) could be interpreted as a deviation in gender assignment rather than agreement, as we have noted. However, Martin otherwise has no deviations in noun inflection, and he also produces the target-like neuter definite inflection of the noun *stålbruk* ‘steelworks’ (17). As we have seen, this is the general pattern among the speakers.

(17)     *stålbruk-et*             (Immigrant Swedish)

           steelworks.n.sg.def (Martin)

Both Annie and Martin can be assumed to have fully acquired the baseline language as children in Sweden, but Martin's earlier and more abrupt onset of English has caused attrition to a higher extent. Among the Heritage speakers that were born in America, early onset of English appears to matter to some extent: Amos and Norman show more deviations than e.g., Arthur, Albert, and Konrad. Amos has also gone the longest without speaking or hearing Swedish. For these speakers, early English generally correlates with less Swedish later in life. It is worth noting that Norman has a high frequency of deviations, despite the fact that he was a monolingual until the age of five, and despite the fact that he is married to Annie (who is clearly more used to speaking Swedish). We return to this briefly in Section Attrition, Acquisition, and Relearning below.

Now, the difference between the speakers is not only how frequent the deviations are, or to what extent the complexity of the noun phrase matters. The speakers also show different patterns with respect to which forms show deviations. We will therefore look a bit closer at the types of deviations.

One weakness with the data is that the different genders are not equally represented: Neuter is much less common than common gender. For instance, Konrad has no complex noun phrases in the neuter, and he produces 74 simple noun phrases with common gender, but only seven with expected neuter. His only deviation involves common gender for neuter. Moreover, it might appear from Table 7 that Norman has little difficulty with simple noun phrases, but all of the examples involve determiners and quantifiers with common gender morphology; the non-target example is the only case with a neuter noun. Also in the complex noun phrases, the deviating examples have common gender forms for neuter. Here, there are, however, also target-like examples with neuter. The fact that Norman produces some examples of neuter morphology suggests that he has some knowledge of the distinction, but our data is unfortunately not conclusive as to whether his use of gender morphology is systematic or not.

From the limited data, we can note that Norman seems to have a tendency to generalize the default^23^. This is also true for a couple of other speakers. All non-target-like examples produced by Martin involve common gender instead of neuter (cf. 16 above). Mostly, it is the indefinite determiner that deviates [*en* for *ett* as in both (16a) and (16b)], but he also has a couple of examples of deviating adjectives [as in (16b)]. Theodor's deviations, too, all involve common gender for expected neuter. Both adjectives and determiners deviate, and to an equal extent. Deviating examples from Theodor are given in (18).

                                                       (Heritage Swedish)

(18)   a.   *en                   flyg*      (target *ett* an.n.sg.indef)

               an.c.sg.indef. airplane.n.sg

               'an airplane' (Theodore)

         b.   *en                 hög                   skratt*

               a.c.sg.indef. high.c.sg.indef. laughter.n.sg

               'a high laughter' (Theodore)

               (target *ett* an.n.sg.indef *högt* high.n.sg.indef)

In fact, Amos seems to be the only speaker that sometimes uses neuter for common gender. All of these examples involve the neuter definite determiner, *det*, as in (19)^24^.

(19)   a.   *det               yngste*

               the.n.sg.def youngest

                 *son* (target: *den* 'the'.c.sg.def) (Heritage Swedish)

                 son.c.sg

               'the youngest son' (Amos)

While Amos's adjectival inflection also deviates from the baseline in a few cases, 7 of his 12 deviations are of the type in (19), involving the prenominal definite determiner. In fact, Amos has no examples of a common gender definite determiner. It seems then that the determiner *det* is unmarked for gender, for this speaker. We can also note that Amos uses the definite determiner *det* in simplex noun phrases (four examples), combining it with the definiteness suffix (20). Here, the target would involve the simple noun in definite form, without the pre-posed determiner. (Note also that double definiteness is missing in (19) above; Double definiteness would here be required in the baseline.)

(20)      *det               båt-en*

             the.n.sg.def boat. c.sg.def

                    (target: *den*   'the'.c.sg.def) (Heritage Swedish)

             'the boat' (Amos)

The pattern with *det* for expected common gender *den* is not found with the other Swedish speakers (but we saw that this happens quite often with the Norwegian heritage language speakers in complex noun phrases). For other speakers, a common gender determiner is used for a neuter determiner, as in the examples in (18a) and (21), but notice that these are indefinite, and hence not competitors with the neuter definite determiner. There are also target-like uses of the common gender determiner, as in the examples in (22).

(21)                                                    (Heritage Swedish)

         a.   en                     *sjukhus* (target: *et* 'a'.n.sg.def)

               a.c.sg.indef hospital.n.sg.indef

               'a hospital' (Arthur)

         b.   *en                barn (target: *et* 'a'. n.sg.def)*

               a.c.sg.indef child.n.sg.indef

               'a child' (Arthur)

(22)                                                 (Heritage Swedish)

               *den                 lilla         byn*

               the.c.sg.indef small.def village.c.sg.def

               'the small village' (Martin)

### Summary on heritage scandinavian

The results show, first, that gender is in place in the overall majority of speakers. This is obvious by looking at the Heritage Norwegian CANS corpus, which has only 12% deviant agreement. Second, there are differences in the frequency of deviations in agreement: some speakers show a high frequency of deviations, other speakers have few or no deviant agreement forms. At least to some extent, the frequency of deviations correlate with the speakers' use of the heritage language after childhood, rather than with the context of acquisition. This is particularly clear since one of the immigrant speakers behave more like the American-born heritage speakers than like the other immigrant speaker. At least for this speaker, attrition rather than incomplete acquisition has affected his production of agreement morphology. Early bilinguals also show more deviations than those that were monolingual Scandinavian speakers until school start. Third, several of the speakers appear to have more problems with complex noun phases than simple ones. It seems that the linear complexity of the noun phrase itself can be a factor behind the deviations. We return to complexity in Section Gender, Agreement, and Declension Class. Fourth, as far as we can observe, the deviations belong to the agreement domain, and gender assignment has not developed into only a declension class. Support for this can be found in the fact that there are no deviations in post-nominal possessives (or in the use of the definiteness suffix), but some deviations in the form of pre-nominal determiners. The fact that agreement is also often in place argues against an analysis in terms of loss of gender. Fifth, the data reviewed so far show no clear tendency of overgeneralization of the masculine in Norwegian, but some Swedish speakers seem to have a tendency of overuse of common gender forms (default). Sixth, there seems to be one form that is overused in both languages (creating deviant agreement), viz. *det* ‘the’.n.sg.def (see further Section Determiners and Adjectives), but in Norwegian this mostly happens in complex noun phrases. Neuter is not otherwise overused in this way. Seventh, nothing in the data suggests that a three-gender system is by itself more vulnerable than a two-gender system, or that feminine gender is particularly vulnerable. Among the Norwegian speakers with the most non-target forms, one has a two-gender system, one a three-gender system. Finally, and importantly, for one phenomenon we do not find inter-individual variation: With a single exception, the form of the definiteness suffix is target-like. This will be discussed further in Section Attrition, Acquisition, and Relearning.

## Results and discussion

The results of our study of Heritage Norwegian and Swedish show that with respect to declension (considering the definiteness suffix) there is no variation. In the production of agreement morphology, on the other hand, the speakers show partly different patterns. In this section, we discuss the patterns further. In section Determiners and Adjectives, we look at the difference between determiners and adjectives. Section Morphological Form and Type of Gender System is concerned with the morphology that shows up in the deviant cases in the three-gender system and the two-gender system. In Section Gender, Agreement, and Declension Class, we discuss the definiteness suffix and the question whether gender is on its way of being reduced to declension class. Section Attrition, Acquisition, and Relearning briefly comments on the issue of attrition vs. acquisition in Heritage Scandinavian.

### Determiners and adjectives

As noted in Section Determiners, Adjectives, and Agreement above, in many varieties that have a three-gender system, the feminine is visible on determiners, but rarely on adjectives (the Norwegian adjective *lita* ‘little’.f.sg.indef is one of very few adjectives that have a distinct feminine inflectional form). Even so, the Heritage Norwegian data reveals no difficulties regarding this gender, and the feminine determiners are generally used where they should be used according to the baseline.

However, we noted above that some speakers have a different agreement pattern in determiners than in adjectives, even disregarding the feminine. This is clear with the Heritage Swedish speaker Amos, who consistently uses the definite determiner *det* in both neuter and common gender contexts. We noted similar cases in Norwegian. An example of each is repeated from (20) and (10), respectively.

(23)   a.   *det                     båten*           (Heritage Swedish)

               the/that.n.sg.def boat.c.sg.def

               'the boat' (Amos)

               (target: *den* 'the'.c.sg.def)

         b.   *det               siste      plass*     (Heritage Norwegian)

               the.n.sg.def last.def place.m.sg.indef

               'the last place' (chicago_IL_01gk)

               (target: *den*. m.sg.def)

From the examples in Section A Closer Look at Two of the Heritage Norwegian Speakers on the two Norwegians and those of American-Swedish Amos, it is clear that substituting other determiners with the neuter *det* ‘the’.n.sg.def is a tendency for some of the speakers (but not all). The fact that this determiner in both languages has the pronunciation /de/ means that it is close both in form and meaning to its English counterpart /ðə/. For Amos, it also clearly lacks gender features, like its English counterpart. This possibly is also compatible with the data from Daisy and Elsa. The indefinite neuter determiner *et/ett* ‘a’ is not overused in this way, cf. (24). (24c, d) are repeated from (10).

(24)   a.   *en                  intressant tur*

               an.c.sg.indef interesting trip.c.sg.indef

                     (Heritage Swedish)

               'an interesting trip' (Amos)

         b.   *ett        par             gånger* (Heritage Swedish)

               a.n.sg. couple.n.sg. time.pl

               'a couple of times' (Norman)

         c.   *en                  butikk*

               a.m.sg.indef shop.m.sg.indef

                     (Heritage Norwegian)

               'a shop' (chicago_IL_01gk)

         d.   *et                  par                      koner*

               a.n.sg.indef couple.n.sg.indef wives

                     (Heritage Norwegian)

               'a couple of wives' (chicago_IL_01gk)

This suggests that the overuse of *det* is an effect of phonological similarity, and thus a transfer of the features of a similar functional lexical item in English. We know from other studies of Heritage Scandinavian that functional vocabulary is often affected by transfer (cf. e.g., Larsson et al., 2015). Lexical convergence due to phonological and syntactic similarity is in fact well-known in multilingual settings (Matras, 2009), and has been applied by Annear and Speth (2015) to understand some features of the Heritage Norwegian lexicon. We saw above that the determiner *det* for these speakers sometimes also occurs in simple noun phrases, where it would not be possible in the baseline. A reviewer points out that this could be an additional argument for transfer from English, since English *the* (unlike the No./Sw. article *det*) is used also with simple nouns. The non-neuter definite article *den* does not occur in simple noun phrases in our data (nor in the baseline). Among the Swedish speakers, Amos is the only one who uses *det* with common gender nouns, and he is also the only one who has unstressed *det* in simple noun phrases^25^. We can observe that transfer is more evident in Amos than in the two Norwegian speakers Daisy and Elsa. Unlike Amos, the latter two have some occurrences of the non-neuter article, and the deviations typically occur in complex noun phrases (see Section A Closer Look at Two of the Heritage Norwegian Speakers)^26^.

We have divided the results in determiners and adjectives in Table 8 below; for Norwegian we only include data from Daisy and Elsa. Overall the frequency of deviations are very similar in the two cases, even if the examples with overgeneralized *det* are included here.

**Table 8:** **Non-target forms of all pre-nominal determiners and adjectives in Heritage Norwegian and Swedish**.

	**Non-target agreement in pre-nominal determiners**	**Non-target agreement in adjectives**
Heritage Norwegian (Daisy and Elsa)	13/89 (15%)	1/15 (7%)
Heritage Swedish	29/318 (9%)	8/72 (11%)
Total	42/407 **(10%)**	9/87 **(10%)**

From Table 8, it appears that the Norwegian and Swedish heritage language speakers are maximally different from each other, for while it is the determiners that present the highest amount of non-target forms amongst the former, it is the adjectives that pose the biggest problems for the latter^27^. This difference can be explained by one of the few syntactic differences between the two languages. While Norwegian possessives are generally post-nominal [see examples in (10) above], Swedish ones are, with few exceptions, pre-nominal. If we had included post-nominal possessives, which are always target-like (see Table 4), amongst the determiners, the difference would have been much smaller.

It should be noted that in our data set, determiners give more opportunity for deviations than adjectives. This need not mean that adjectives are intrinsically easier, but rather that there are only two gender-relevant adjectival inflections, –Ø and –t, and this difference is only visible in the indefinite paradigm (as in *stor* ‘big.m/f.sg.indef’ and *stort* ‘big.n.sg.indef’). Thus, in order to get a neuter form of the adjective, three criteria must be satisfied: (1) The noun must be neuter, (2) the noun phrase must be indefinite, and (3) the noun phrase must be singular. Since we also know that neuter nouns are outnumbered by M/F nouns with a factor of 1:4, there will be very few relevant hits in any corpus. In all the indefinite cases of masculine and feminine, the bare stem form would be used. In the definite form and in the plural, there is only one regular form: the –*e* and –*a* (Norwegian and Swedish, respectively). The small number of non-target adjectives could be due to this. We therefore do not want to conclude that either determiners or adjectives overall pose more of a challenge to heritage language speakers.

However, from acquisitional studies, we might have expected the heritage speakers to show more difficulty with determiners. In studies of the acquisition of agreement, it has sometimes been noted that determiners cause more difficulty than adjectives. In a study of young (2;7-3;3) Norwegian monolinguals and Norwegian-English bilinguals, Rodina and Westergaard (2013) show that determiners can be unspecified for gender. Other studies, too, have shown that the inflection of determiners might be particularly difficult (see e.g., Cornips and Hulk, 2008 on Dutch). From previous work on Heritage Scandinavian (see e.g., Larsson and Johannessen, 2015a,b), we know that our heritage speakers sometimes show the same type of patterns as language learners, and we can attribute this to limited input and incomplete acquisition (cf. the introduction). While this might be the source of transfer of functional vocabulary (as for *det* above), it cannot account for the other deviations. As noted, the Swedish immigrant speaker Martin shows deviations, although he presumably acquired Swedish fully in Sweden, before the time of emigration. If, as we argue, the deviations are rather due to attrition and processing difficulty in the adult speakers, it is less clear that the difference between agreeing forms of functional and lexical vocabulary would matter.

### Morphological form and type of gender system

For determiners, we saw that Amos and some speakers in the Norwegian corpus used the neuter form *det* /de/ also in common gender/masculine contexts. In other cases, we could observe that the deviant forms most often involved generalization of what we have taken to be the default (masculine or common gender). This pattern appeared to be stronger for Heritage Swedish than for Norwegian. In Table 9 below, we give the number of non-target uses of neuter, masculine/common gender, and for Norwegian the feminine (which is only marked on determiners).

**Table 9:** **Gender forms in non-target agreement contexts in Heritage Norwegian (two speakers) and Swedish**.

	**Non-target neuter**	**Non-target common gender/masculine**	**Non-target feminine**
Heritage Norwegian	7/14 (50%)	4/14 (29%)	3/14 (21%)^28^
Heritage Swedish	7/37 (19%)	30/37 (81%)	(Not applicable)
Total	14/51 **(27%)**	34/51 **(67%)**	3/51 **(6%)**

Feminine inflection generally only on determiners.

Non-target neuter can be found in 50% of the Norwegian non-target forms, and 19% of the Swedish ones. These are substantial numbers, but at the same time the difference between the two languages is quite big, and it also turns out that the difference between the speakers is substantial. In fact, all of the Swedish examples where neuter is used for common gender come from the same speaker, Amos. All but one of the Norwegian examples come from Daisy. This suggests that there are individual strategies that are not shared by all the speakers. The loan-transfer of the English determiner *the* to the Norwegian determiner *det* based on similarities in phonological form and syntactic function is one such strategy, as noted above.

Given the arguments for the masculine as a default gender, based on, *inter alia*, the frequency of masculine nouns and their basic phonological form (see Section Gender), it is to be expected that the masculine gender is overused relative to the baseline by heritage language speakers. In the study by Rodina and Westergaard (2013) on monolingual and bilingual acquisition, for instance, the majority of errors are overgeneralization of the masculine. Rodina and Westergaard suggest that frequency of forms might be a relevant factor in the acquisition of gender—children overgeneralize the most frequent form (the masculine) in the input. We have not found a clear tendency for all speakers to generalize the masculine, but there is a substantial number of examples (81% in Heritage Swedish and 29% in Heritage Norwegian). Since, the generalization affects determiners as well as adjectives (which take -Ø in the M/F), it cannot exclusively be accounted for in terms of loss of morphological marking.

We have seen several strategies amongst the speakers, but we have not seen anything that suggests a particular pattern based on whether a speaker has a two- or three-gender system. The non-target forms are not completely random, but are in general related to (1) lexical convergence for the neuter singular definite determiner, and (2) overgeneralization of the default masculine/common gender.

### Gender, agreement, and declension class

There is, as noted, variation in the production of agreement morphology in Heritage Scandinavian, but there is no corresponding variation in the form of the definiteness suffix. The use of the definiteness suffix is target-like (with one single exception). As noted, similar patterns have been observed for child language. For instance, Rodina and Westergaard (2013) show that in child language, agreement is more error-prone than noun inflection. It has sometimes been suggested that the definiteness suffix is rote learned, i.e., learned as a chunk together with the noun (see e.g., Andersson, 1992, p. 183 and cf. Bohnacker, 2003 for a different view). While this cannot be completely ruled out for individual items, or perhaps for individual speakers (e.g., Amos, who combines the definiteness suffix with a pre-posed definite determiner), we do not see that rote learning can fully account for the target-like definiteness suffix. Firstly, speakers clearly alternate between indefinite and definite forms: Even Amos produces both forms like *hus* ‘house.indef’ and *hus-et* ‘house.n.sg.def’. Definite forms sometimes occur with what appears to be productive formations, and in code-switching contexts. For instance, Norman uses the form *korporejt-en* ‘corporation.c.sg.def’, and Daisy *river-en* ‘river.m.sg.def’. Secondly, no speaker attaches plural morphology to the definite form, but the speakers always correctly place plural morphology closer to the root than definiteness morphology (e.g., *kusin-er-na* ‘the.cousins.pl.def’, not ^*^kusin-en-er, cf. Bohnacker, 2003). One would have expected at least some examples of plural morphology following a singular definite noun if the latter were treated as a chunk. There is therefore at least some tentative evidence that the speakers do analyse the definite forms of nouns, and do not simply treat them as chunks. Thirdly, and importantly in the present context, the definiteness suffix seems to be acquired early by monolingual children in Norway and Sweden (cf. Bohnacker, 2003; Rodina and Westergaard, 2013 and references there), and it is likely to have been fully acquired by the heritage speakers in the present study; this is also what our results suggest. With respect to gender, the Scandinavian heritage speakers thus have a clear advantage over L2 learners, for whom gender assignment is difficult (see e.g., Andersson, 1992; cf. Montrul et al., 2012, 2014). Moreover, it seems that the definiteness suffix is not affected by attrition. If the heritage speakers can access the noun, they can also access its declension class and (perhaps) its gender.

As far as we can see, there are at least two ways of interpreting the difference in behavior with respect to the suffix and agreement morphology, taking gender to be a lexical category of the noun that is visible in agreement morphology on adjectives and determiners. One possibility is that the gender system is unstable in Heritage Scandinavian, and that what we have interpreted as gender in the definiteness suffix has been (or is on its way to be) reduced to pure declension class. We know from other studies of heritage language that gender systems can be vulnerable (see e.g., Montrul et al., 2008; Polinsky, 2008). The other possibility is that gender is in fact stable in Heritage Scandinavian, and that the variation is more superficial, with the cases of non-target-like agreement being production errors. In the former case, we expect the deviations in agreement to be systematic, and possibly follow the patterns we know from historical changes in the gender system of Scandinavian (e.g., that feminine disappears, or that gender is maintained longer in determiners than adjectives). In the latter case, we expect the type of task and the processing difficulty to be factors, and we expect the behavior of the heritage speakers to be more inconsistent.

We believe that the gender system in Heritage Scandinavian is overall stable. Firstly, most speakers show no or few deviations in agreement (regardless of whether they have a two- or a three-gender system). Secondly, we cannot see that determiners maintain gender distinctions to a higher degree than adjectives (or the other way around).

Thirdly, we see from Table 4 in Section Agreement in the Corpus of American Norwegian Speech that complexity is important for the Norwegian heritage speakers. In complex noun phrases Daisy and Elsa have 50% non-target forms, while the number is reduced to 8% for the simpler adjective–noun combinations, and down to 0% in the simplest noun phrases (with post-posed possessives). The Swedish heritage speakers have 16 and 7% for the most complex and the least complex ones (see Table 7, Section Gender Agreement in Complex and Simple Noun Phrases). The individual differences are big, and might account for the difference between the two groups. However, for the speakers that show deviations to a higher degree, it seems that the task of applying the same gender morphology to several items in a noun phrase is the biggest problem.

Importantly, complexity here does not necessarily mean structural (syntactic) complexity. Following Julien (2005) and others, we can assume that structures with post-nominal possessives are structurally more complex than structures with pre-nominal possessives (the former involving movement of the head noun). As shown by Anderssen and Westergaard (2010), monolingual Norwegian children seem to prefer the syntactically less complex order with pre-nominal determiner and use it also in contexts where it is not used in the input (where the post-nominal determiner is more frequent). Based on this and other evidence, they argue that structural complexity, rather than frequency determines the path of acquisition. In a later study (Westergaard and Anderssen, 2015), they show that the same does not necessarily hold among Norwegian heritage speakers, who rather overuse the post-nominal possessive. They conclude that structural complexity is not a factor for attrition in the same way as it is in acquisition.

With respect to agreement morphology, too, prenominal determiners appear to be more difficult than post-nominal possessives. Instead of structural complexity, it seems that the linear distance between agreeing form and head noun matters for our speakers. This suggests that the speakers generally have an intact gender system, which they fail to adhere to in situations that are demanding for their working memory. Myles (1995) makes a similar case for second language acquisition in French, where she suggests that agreement morphology is sensitive to level of embedding. She argues that the degree of automatization in processing is crucial; only when low-level processes (processing in local domains) are automatic, is the short-term memory freed and can deal with higher-level processes (cf. e.g., Pienemann, 1998 and many others on processability).

More recent studies have confirmed Myles (1995) results, but there is some disagreement as to the reason behind the difficulties (see Keating, 2009; Foote, 2011 and references there). For Myles, the relevant factor in second language acquisition is structural complexity. As pointed out above, in our study it rather seems to be linear distance that matters. This is more in line with results like those in Keating (2009), who shows with an eye-tracking experiment that while advanced learners of Spanish have acquired gender distinctions, they are non-native-like by being affected by the distance between nouns and modifiers^29^. The fact that our heritage speakers have not used the language regularly for many years clearly has effects on the burden on their short-term memory and processing abilities. For instance, lexical retrieval is known to become less automatic (and more costly) in attrition (Polinsky, 2008), and gender is clearly tied to lexical retrieval.

Our conclusion is that the underlying Scandinavian gender system is not particularly vulnerable, so that the definiteness suffixes of the nouns, which are always correct, actually signify gender and not just declension class. At the same time we see some signs of vulnerability amongst the most attrited speakers, where we find both lexical convergence of the definite determiner *det* /de/ with English *the* /ðə/, and a (somewhat weak) tendency to generalize the masculine/common gender.

Thus, our result is in contrast with previous studies of other heritage languages, where gender as noted has been shown to be vulnerable (e.g., Polinsky, 2008; Montrul et al., 2008, 2014). For instance, in a study of Spanish gender, Alarcón (2011) concludes that gender assignment (rather than agreement) is particularly sensitive to incomplete acquisition, and Polinsky (2008) observes systematic changes in the gender system of Heritage Russian. For Heritage Scandinavian, on the other hand, gender agreement appears to be more sensitive than gender assignment, and there are no systematic changes in the gender system (again, if we disregard *det* in individual speakers). The question, then, is why Scandinavian would be different.

Now, we expect that some of the more systematic changes in gender systems in heritage languages are due (to a higher extent) to incomplete acquisition, rather than attrition. In fact, it is possible that the differences between e.g., Spanish and Russian heritage language, on the one hand, and Heritage Scandinavian, on the other, stem from differences in the acquisitional process. As pointed out by Bohnacker (2003), studies of L1 acquisition of Swedish (e.g., Plunkett and Strömqvist, 1992; Andersson, 1992) suggest that gender is acquired early and with ease, in contrast with acquisition of gender in many other languages. This appears to be the case even if Scandinavian gender is largely unpredictable from phonology and semantics. One possible explanation, suggested by Andersson (1994), is that the evidence for the gender of a noun in Swedish is not only found in agreement patterns, but also in the definiteness suffix, which unambiguously signals the gender of the noun (cf. the discussion above). Thus, the acquisition of gender can go hand in hand with the acquisition of declension class. We thus hypothesize that the vulnerability of gender in other heritage languages could stem from incomplete acquisition, though the deviations we have noted in the present study are largely a consequence of attrition.

However, if this is on the right track, and the cross-linguistic differences correlate with differences in the acquisition of gender (due to the varying evidence for gender), one might expect a clear difference between Heritage Swedish and Heritage Norwegian, contrary to what we find. Recent studies of acquisition of gender in Norwegian (Gagliardi, 2012; Rodina and Westergaard, 2013, 2015) have argued that gender assignment (not agreement) is in fact difficult for children. Particularly the feminine gender appears vulnerable, and deviations from adult language might persist well into school age. (There are also deviations in the use of neuter, but to a lesser degree; Rodina and Westergaard, 2015, p. 176). It is possible that this difference between Swedish and Norwegian is due to the different gender systems (two vs. three genders), but it is also likely that the linguistic situation in Norway has something to say. Norwegian children are typically exposed to more than one gender system, since one of the standard varieties (Bokmål) can have a two-gender system (or perhaps a few remnants of the feminine gender), whereas most dialects have three genders. On the basis of data from different generations of speakers of the Tromsø dialect, Rodina and Westergaard (2015) in fact argue that the gender system is changing, and that the feminine gender appears to be on its way out. In addition to the bi-dialectal situation, they point to independent changes that lead to the loss of some of the morpho-phonological cues for the feminine (2015:181). Moreover, in the adjectival inflection, the feminine gender is as noted only rarely distinguished from the masculine. Crucially, the situation for the Norwegian heritage speakers is quite different, since there is no influence from the standard language (cf. Johannessen and Laake, forthcoming). Instead, the heritage speakers generally only speak and understand their own dialect, and they have no knowledge of the written language. It is therefore possible that the situation for the Norwegian heritage speakers is more similar to that of the Swedish speakers. However, some caution is required in the interpretation of the results, since the studies of acquisition in Norway focus on particular dialects, since the individual variation among the heritage speakers is considerable, and since the different studies employ partly different methodologies. Additional work is clearly required.

### Attrition, acquisition, and relearning

The deviations in agreement seem to be a consequence of language attrition at the level of the individual. The speakers in the present study are old (with the exception of Martin, 55 years old at the time of the recording) and have not spoken Scandinavian daily for many years. The number of deviations does not necessarily correlate with acquisitional context. Most of the speakers that show no deviations are born in the USA, and one of our two immigrant speakers show agreement deviations.

At the same time, it seems that the onset of the dominant language (English) is an important factor. As noted for Swedish, the speakers with the highest number of deviations were all early bilinguals. This is also true for the Norwegian speaker Daisy. A more typical pattern is acquisition of the majority language at school start.

From the perspective of early bilingualism and language use later in life, it is perhaps surprising that the heritage speakers Norman (Swedish) and Elsa (Norwegian) show a relatively high number of deviations. Norman reports that he was monolingual until the age of five, and he is married to a Swedish immigrant. He reported that he started speaking English with his parents before school start, in order to prepare for school, and it seems that his connections to the Swedish community had mostly been through his wife, who he met when he was around 20 years old. Elsa also has had a lot of contact with Norway at an adult age, given that two of her children have married there. It is possible, we think, that the deviations for both Elsa and Norman can to some extent be a consequence of language loss followed by relearning. It is possible that relearning makes the heritage language less native-like, and perhaps even L3-like. Polinsky (2015a) shows that, in an environment of instruction of the written baseline language, heritage speakers do outperform L2 learners in the perception and production of phonology. However, when learning grammatical features, heritage speakers were outperformed. Viswanath (2013, p. 39) further shows that heritage speakers over-regularize forms in a learning context. It seems, therefore, that relearning does not necessarily improve the heritage speakers' competence. The fact that Norman and Elsa have had close encounters with the European Scandinavian varieties as adults might have had the same effect as relearning in a formal context.

One difficulty in their relearning is that the relearnt language (the modern Scandinavian dialects/standards) is in fact substantially different from their original heritage language (compare Polinsky, 2015a). Further studies on heritage language and relearning are required.

## Conclusion

In this paper, we have looked at agreement in the noun phrase. The Scandinavian noun phrase clearly poses several difficulties in language acquisition, including double definiteness marking (Anderssen, 2012, p. 4), agreement and gender assignment to the noun, and it has previously been shown to be affected in attrition.

To sum up our investigation, the results show the following. First, gender is in place in the overall majority of all our speakers. Among the 34 speakers in the Heritage Norwegian CANS corpus, there is only 12% deviant agreement with respect to the baseline, and among the eight Heritage Swedish speakers only 10% deviance. Second, there is considerable variation among speakers. Nearly 60% of the Norwegian speakers show no deviant forms, while a few speakers show a considerable number. Of the Swedish Heritage and Immigrant speakers, five speakers have deviance in 0–9% of their total number of noun phrases, while three have 19–28%. Third, we see that the complexity of the noun phrase matters: The two most attrited Norwegian speakers have 50% deviance in complex noun phrases, and 0–8% in various kinds of simple noun phrases. The Swedish speakers have 16% deviance in complex noun phrases and only 7% in simple noun phrases. This suggests that the deviations are due to processing difficulty. Fourth, the deviations belong to the agreement domain, and not in the definiteness suffix. It can be concluded that gender assignment is largely in place, and that the definiteness suffix has not developed into a marker of just declension class. Support for this can be found in the fact that there are no deviations in the Norwegian post-nominal possessives (or in the use of the definiteness suffix), and only a few deviations in the form of pre-nominal determiners, in both languages. Fifth, the data show that there is a tendency to overgeneralize the masculine (which is the default gender), but there is also one particular neuter form which is overused (creating deviant agreement), probably due to its similarity in form and meaning to its English counterpart, viz. *det* /de/, ‘the’.n.sg.def, from English, similar to *the* /ðə/ def. No other forms of neuter are overused in this way, clearly showing that this is an effect of lexical convergence. Finally, nothing in the data suggests that a three-gender system is by itself more vulnerable than a two-gender system, or that feminine gender is particularly vulnerable. Among the Norwegian speakers with the most non-target forms, one has a two-gender system, one a three-gender system. The patterns we can observe in Norwegian three-gender speakers are also found among the Swedish two-gender speakers.

We would like to point out that the data we have used come from a variety of speakers (Norwegian and Swedish, heritage and immigrant) and sources (in depth studies of interviews and automatic counts of a large corpus), and that this has given us the possibility to investigate both general patterns and inter-speaker variation, and to explore different types of explanations. Going back to the factors that have previously been shown to affect the properties of Heritage Scandinavian, we can note the particular acquisitional context of the American-born heritage speakers do not necessarily affect gender agreement. Moreover, there is overall very little evidence of transfer from English (with a single exception). We do not see a general simplification of the gender system across Heritage Scandinavian. Instead, we have considerable inter-individual variation. We have observed that one immigrant speaker show more deviations than many of the American-born heritage speakers. For that reason, among others, we have wanted to argue that the deviations we find are due to attrition. Given previous studies, this is perhaps not surprising: Morphology has been shown to be sensitive in attrition. As expected, it appears that the time of onset of English matters for the degree of attrition, in combination with the use of the L1 later in life. However, several factors are clearly intertwined, and they call for further study; for some speakers, relearning might be involved, as well. For some individuals, there are specific deviations that might also be due to reanalysis in the first language acquisition. This would be one way of accounting for the lexical convergence of one determiner. Notably, this change is restricted to a single functional word.

### Conflict of interest statement

The authors declare that the research was conducted in the absence of any commercial or financial relationships that could be construed as a potential conflict of interest.

## Footnotes

^1^We use the term *heritage language* in the narrow sense to refer to a language acquired as a first language in a naturalistic setting, but in a society where it is not the dominant language. For heritage speakers, the first language will generally not continue to be the strongest, primary language after school start (cf. e.g., Polinsky, 2008 and references there). *Heritage Scandinavian* is here used to cover Heritage Norwegian and Heritage Swedish in America. For present-day Heritage Scandinavian speakers in America, English is always the primary language, and Scandinavian is weaker, secondary, and used only in a restricted set of situations and among a limited group of speakers (typically family members).

^2^The term *incomplete acquisition* has caused some debate (see e.g., Pascual y Cabo and Rothman, 2012), and not everybody agrees that the two processes that the terms *incomplete acquisition* and *attrition* cover should be distinguished. See Larsson and Johannessen (2015b) for additional discussion.

^3^The data used in this paper is all from recordings done in the American Midwest in the 2010s. The recordings include naturalistic speech, with no elicitations, only recordings of sociolinguistic interviews between speakers and researchers, or conversations between speakers. Many of the Norwegian recordings have been transcribed and digitally processed, and are now available in a searchable corpus, Corpus of American Norwegian Speech (CANS), see Johannessen (2015b). At the time of the present study, the corpus contains the transcriptions, audio and video of the speech of 34 speakers, approximately 120,000 words. The Swedish recordings include both Heritage Scandinavian speaking descendants of the early immigrants, who are typically over 80 years old, and younger speakers that are descendants of more recent immigrants. A few speakers that emigrated themselves, and who we can refer to as immigrant speakers, were also recorded (see Andréasson et al., 2013 for an overview of the methodology); in the following we refer to their language as *Immigrant Swedish*. Only a few of the Swedish recordings have been transcribed, and they have not been included in the corpus. To establish the baseline, we have used older recordings of American Norwegian, collected by Einar Haugen in the 1940s–50s, and recordings of American Swedish collected by Folke Hedblom and Torsten Ordéus in the 1960s. For an overview of the older recordings, see Johannessen and Salmons (2012, p. 139) and Andréasson et al. (2013).

^4^See Sorace (2004) for a comment on this potential methodological problem. In the present study, this is not a problem, we believe. Firstly, the deviations from the established baseline are not shared across the community. Secondly they show up even among present-day emigrant speakers, but they do not occur among L1 speakers in Norway or Sweden, nor in the older recordings of immigrant speakers.

^5^Norwegian has two official written standards, *Bokmål* and *Nynorsk*, and we have chosen to follow the former in the examples.

^6^Some varieties also have apocope, and drop the final vowel in some contexts (see e.g., Dahl, 2015, p. 135), but this is irrelevant for our study, which only looks at the gender agreement marking, in which a final vowel exponent is never an option.

^7^The opposite is also true—there are Scandinavian varieties that show considerable reductions in the nominal declension system, collapsing the feminine and the masculine classes, but which still show evidence of a three-gender system in pronouns and possibly determiners. See Davidson (1990) for a discussion of the historical development in Swedish.

^8^Heggstad simply counted the frequency of each gender in a dictionary for Bokmål Norwegian, and found 54% masculine nouns, 21% feminine nouns and 25% neuter nouns.

^9^Trønder Norwegian is a cover term for the dialects spoken in the Trøndelag area and its surroundings in the middle part of Norway.

^10^Teleman (1969) argues against treating common gender as the default in Swedish, on the basis of the distribution of neuter singular morphology, which shows up in the absence of agreement in Swedish and Norwegian, as in other Indo-European languages. Teleman therefore concludes that it is the default. However, neuter forms do not seem to be the default spell-out of gender features, but rather, the default in the absence of gender features. We therefore maintain that masculine is the default value of gender features, and that -Ø is the elsewhere suffix for adjectival agreement.

^11^Again, this connection between gender and declension class does not hold for all varieties, and in some varieties the situation is changing, as discussed by Lødrup (2011) and Rodina and Westergaard (2015). As far as we know, the distinction between neuter and non-neuter is however maintained in the definiteness suffix.

^12^We do not include double definiteness marking in our study of agreement. One common analysis of double definiteness is to treat the prenominal determiner as a placeholder (e.g., Delsing, 1993), but it is also sometimes assumed that the two definite markers contribute partly different features (Julien, 2005). In other words, a noun phrase will here be considered target-like if double definiteness is missing, as long as the gender agreement is right. A few examples with modifiers in the neuter singular with plural nouns have been excluded from the study; neuter singular is sometimes used for mass nouns, or to mark absence of individuation (see e.g., Josefsson, Submitted). For instance, the Swedish speaker Annie produces examples like (i). (i)         *mycket      finsk-or*            much.n.sg Finnish.woman.pl            'many Finnish women' This is grammatical in many varieties of Swedish and Norwegian, given that the women are not individuated.

^13^We do not have full transcriptions for all of these speakers yet, so we also use the recordings. A few unclear examples have been disregarded.

^14^Following Johannessen (2008, p. 185–186), one could assume that there is no syntactic difference between the pre-posed article, the pre-posed possessive and the demonstrative in Norwegian; they are all determiners. Johannessen argues further that there seems to be individual variation between a system like the Norwegian one, and that of Danish, in which the demonstrative is not a determiner. This is contra Julien (2005) and Leu (2015), who argue that the proposed article, possessives and demonstratives have partly different syntax. Our data is too limited for us to investigate the behavior of the different elements in any detail, and for simplicity we refer to all three groups as determiners. For the present purposes, the particular analysis is not important, since it seems clear that what is relevant here is not the syntactic complexity of the noun phrase, but *linear* complexity (see further Section Gender, Agreement, and Declension Class).

^15^When her maternal grandmother came from Norway to live with them, Daisy was 7. They spoke only Norwegian together. Daisy's Norwegian is definitely the dialect of her mother and maternal grandmother, originating in the town of Moss, Østfold.

^16^Both speakers also produce target-like complex noun phrases: (i)     den                store    bygning         the.m.sg.def big.def building.m.sg         'the big building' (chicago_IL_01gk) (ii)   den                  eldste         jenta        the.m/f.sg.def oldest.def girl.f.sg.def        'the oldest girl' (harmony_MN_02gk)

^17^This example does not have the structure det+adj+noun, since the noun is appositive here. It is included, though, since there is a noun that determines the gender here, even though it is not expressed in the narrow noun phrase.

^18^One reviewer asks if maybe the determiner is targeting the noun *arbeid*, and asks what a monolingual corpus might show. We have searched for the non-target *det slags* and the target *den slags* in *Leksikografisk bokmålskorpus*, and found 1–one– example of *det slags*, but 1477 examples of *den slags*. It is clear that targeting a different noun here is not something that is done in monolingual language use.

^19^We choose to count that as non-target here, but this judgment is uncertain. Elsa's Norwegian ancestors are from the East of Norway (Østerdalen, Gudbrandsdalen, Ringerike, and Trøndelag), and in many of these dialects an unstressed neuter indefinite article is pronounced /ei/ or /i/, like the feminine article, instead of the stressed (and more standard) /et/. A relevant search in the Nordic Dialect Corpus (Johannessen et al., 2009) shows that this is the case in *inter alia* Kvam, Gausdal, Nordre Land, Brandbu, Åsnes, Drevsjø, Tolga, Røros, Dalsbygda, Gauldal, Meråker and Inderøy, which are all in the areas where she has ancestors. She never uses the article /et/. The speakers from the Norwegian areas mentioned all vary between a variant with –t, /et/ and one without, /ei/ or /i/.

^20^From (13b) it looks as if Daisy has retained the feminine gender after all. As pointed out by Fretheim (1976/1985) and Lødrup (2011) post-nominal possessive forms are special since they can retain the old feminine morphology although the three-gender system has otherwise disappeared. Lødrup shows that these forms should not be treated as feminine agreement forms in modern varieties of Oslo Norwegian. Whether his analysis can be extended to speakers like Daisy, or if the form *mi* shows that Daisy has some remnants of the three-gender system is not clear from the available data.

^21^The postposed possessives in (13) show that the heritage speakers have a good command of another part of the grammar, too. Examples (13a,d) show that they are aware that certain kinship terms (especially *mor* ‘mother’, *far* ‘father’ and *bror* ‘brother”) can occur with postposed possessives with an indefinite form of the noun, unlike other nouns, as in (13b,c).

^22^Some speakers have the definiteness suffix -*a*, in addition to -*en*. For instance, Konrad uses both the form *boken* and the form *boka* for ‘the book’. This is not unexpected, since it is common in many Swedish dialects (as it is in Norwegian). The distribution is targetlike. Both -*a* and -*en* are restricted to common gender nouns. There is no evidence for feminine agreement in the language of Konrad.

^23^The conclusion is strengthened by examples with predicatives, where common gender is also overgeneralized, and where we, in fact, find no cases with neuter morphology.

^24^There are also examples of neuter for common gender in Amos' written production, and these examples are of a different type. They have neuter also on adjectives and other types of determiners than the preposed definite article: (i)     a.    *en                rolig-t              jultid*               a.c.sg.indef fun.n.sg.indef christmas.time.                         (target: rolig 'fun'. c.sg.indef)                         c.sg.indef               'a fun Christmas time' (Amos)         b.   *ett               ny-tt                flickvän*               a. n.sg.indef new.n.sg.indef *girlfriend*. c.sg.indef               'a new girlfriend' (Amos)               (target: en 'a'.c.sg.indef, ny 'new'. c.sg.indef) Amos does not otherwise treat vän ‘friend’ as a neuter noun (in his written or oral production); cf. (ii) which shows target-like agreement. (ii)     *en                vän*         a.c.sg.indef friend. c.sg.indef             'a friend' (Amos)

^25^Demonstrative *den/det* (distinguished as it carries stress) is however used in simple noun phrases, as in the baseline language.

^26^A reviewer asks whether the determiner *det* (which in the baseline only occurs in complex noun phrases) does not interfer with the complexity factor. Since only one of the Swedish speakers shows this pattern, and since he uses *det* in both simple and complex noun phrases (to what appears to be an equal extent), this does not affect the overall difference between complex and simple noun phrases. The same seems to be true for Norwegian, where the extended *det* is only present with a limited number of speakers. As noted, above, also complexity appears to matter to a varying extent for different speakers.

^27^It should be mentioned that the two speakers do produce several non-target adjectival inflections, but these are related to definiteness and number, and not gender.

^28^But recall that from Section A Closer Look at Two of the Heritage Norwegian speakers that two of the determiners categorized in this cell might actually be target neuter, so this will not be discussed further.

^29^Also other studies show that there is some evidence for so-called shallow processing in second language learners (see e.g., Sorace, 2006 for discussion). In the context of gender agreement Keating (2009) operationalizes shallow processing “in terms of the distance that separates agreeing constituents.”
